# EPR Oximetry of Cetuximab-Treated Head-and-Neck Tumours in a Mouse Model

**DOI:** 10.1007/s12013-017-0814-5

**Published:** 2017-07-29

**Authors:** H. Gustafsson, A. Kale, A. Dasu, A. Lund, P.-H. Edqvist, K. Roberg

**Affiliations:** 1 Department of Radiology Norrköping and Department of Medical and Health Sciences (IMH), Linköping University, Linköping, Sweden; 2 Center for Medical Image Science and Visualization (CMIV), Linköping University, Linköping, Sweden; 3 Division of Oto-Rhino-Laryngology and Head and Neck Surgery, Department of Clinical and Experimental Medicine, Linköping University, Linköping, Sweden; 4 Department of Medical and Health Sciences, Linköping University, Linköping, Sweden; 5 The Skandion Clinic, Uppsala, Sweden; 6 Department of Physics, Chemistry and Biology (IFM), Linköping University, Linköping, Sweden; 7 Department of Immunology, Genetics and Pathology and Science for Life Laboratory, Rudbeck Laboratory, Uppsala University, Uppsala, Sweden

**Keywords:** EPR oximetry, Tumour oxygen pressure, Head and neck cancer, Cetuximab, Hypoxia, Metabolism

## Abstract

Head and neck squamous cell carcinoma (HNSCC) tumours are associated with high mortality despite advances in therapy. The monoclonal antibody cetuximab (Erbitux^®^) has been approved for the treatment of advanced HNSCC. However, only a subset of HNSC patients receiving cetuximab actually responds to treatment, underlining the need for a means to tailor treatments of individual patients. The aim of the present study was to investigate the effect of cetuximab treatment on tumour growth, on tumour partial oxygen pressure as measured by LiPc electron paramagnetic resonance oximetry and on the expression of proteins involved in tumour growth, metabolism and hypoxia. Two HNSCC cell lines, UT-SCC-2 and UT-SCC-14, were used to generate xenografts on female BALB/c (nu/nu) nude mice. Mice with xenografts were given three injections of intraperitoneal cetuximab or phosphate-buffered saline, and the tumour volume was recorded continuously. After treatment the tumour partial oxygen pressure was measured by LiPc electron paramagnetic resonance oximetry and the expression of epidermal growth factor receptor (EGFR), phosphorylated EGFR, Ki-67, MCT1, MCT4, GLUT1, CAIX and HIF-1α were investigated by immunohistochemistry. In xenografts from both cell lines (UT-SCC-2 and UT-SCC-14) cetuximab had effect on the tumour volume but the effect was more pronounced on UT-SCC-14 xenografts. A higher tumour oxygenation was measured in cetuximab-treated tumours from both cell lines compared to untreated controls. Immunocytochemical staining after cetuximab treatment shows a significantly decreased expression of EGFR, pEGFR, Ki67, CAIX and nuclear HIF-1α in UT-SCC-14 tumours compared to untreated controls. MCT1 and GLUT1 were significantly decreased in tumours from both cell lines but more pronounced in UT-SCC-14 tumours. Taken together, our results show that cetuximab treatment decreases the tumour growth and increases the tumour partial oxygen pressure of HNSCC xenografts. Furthermore we found a potential connection between the partial oxygen pressure of the tumours and the expression of proteins involved in tumour growth, metabolism and hypoxia.

## Introduction

Head and neck squamous cell carcinoma (HNSCC) is a malignancy that is associated with high mortality despite advances in therapy. The treatment of HNSCC is heavily based on radiotherapy in combination with surgery and/or cytostatic drugs. However, radiotherapy and/or chemotherapy resistance and tumour recurrences are important clinical problems in the management of HNSCC.

The epidermal growth factor receptor (EGFR) is a transmembrane cell surface receptor found mainly in cells of epithelial origin. In malignant tumours EGFR overexpression is linked to poorer prognosis [1, 2]. Approximately 80% of HNSCC exhibit an increased expression of EGFR making it a suitable candidate for targeted therapy. The activation of EGFR promotes angiogenesis, cell migration, survival, invasion and proliferation. The monoclonal antibody cetuximab (Erbitux^®^) has been approved for the treatment of advanced HNSCC. Studies have shown an increase in overall survival of almost 20 months in HNSCC patients who received targeted therapy against EGFR in combination with radiotherapy compared to radiotherapy alone [3]. At the same time only a subset of HNSCC patients receiving cetuximab actually responded to treatment, underlining the need for a means to tailor treatments to individual patients [3, 4].

Hypoxia, which is very common in solid tumours, is associated with treatment resistance, increased invasion and poor clinical outcome. Treatment success rates for HNSCC are known to be correlated to tumour oxygenation with low treatment success rate associated with tumour hypoxia, and thus a number of clinical studies have reported that the amount and the severity of hypoxia correlate with poor prognosis from radiation therapy [5–8]. Accurate determination of tumour hypoxia is therefore a highly relevant issue since it could show the evolution of tumour oxygenation during the treatment and could also identify the patients in need of more aggressive therapy approaches to counteract the radioresistance induced by hypoxia.

In the light of the importance of hypoxia in radiation therapy, several methods have been proposed to quantify tumour oxygenation [9]. Invasive polarographic electrodes determining oxygen concentration from the current resulting from an electrochemical reaction are considered a gold standard for measuring tumour hypoxia in vivo [10], although theoretical simulations showed that they only give a qualitative characterisation [11, 12]. Other successfully used methods involve the use of nitroimidazole compounds that are preferentially metabolised in hypoxic conditions and are imaged either ex vivo in case of fluorescence labelling [13] or in vivo for radiolabelled compounds like fluoromisonidazole, FETA (fluoroetanidazole), fluoroazomycin arabinoside and a number of others [14–18]. Other methods like blood oxygen level-dependent magnetic resonance imaging [19] give information on the vascular oxygenation which could be quite different from tissue oxygenation [9].

A less explored method in the array of measuring techniques is electron paramagnetic resonance (EPR) oximetry that holds a number of advantages. EPR is a spectroscopic method for studies of paramagnetic species, such as radicals and transition ions [20], and EPR oximetry is based on the paramagnetic nature of the molecular oxygen. In EPR oximetry the partial pressure of oxygen can be measured non-invasively in vivo by means of a direct relationship between the EPR line width of a paramagnetic probe present in the tissue of interest and the oxygen pressure [21, 22]. EPR oximetry is currently evolving towards clinical use both in the form of low frequency in vivo EPR spectroscopy [23] and in vivo imaging (electron paramagnetic resonance imaging, EPRI) [24]. EPR oximetry provides direct quantitative measurements and/or images of the oxygen partial pressure (pO_2_) in, e.g., mm-Hg in the tissue of interest (e.g., tumour), and it has been shown that the correlation between EPRI oximetry and, e.g., oxylite measurements is good both in terms of the spatial distribution pattern and the pO_2_ magnitude [25]. For these reasons, EPR oximetry is being developed towards clinical use [26–28].

The aim of the current study was to investigate the effect of cetuximab treatment on tumour partial oxygen pressure as measured by lithium phthalocyanine (LiPc) EPR oximetry, on tumour growth, and on the expression of proteins involved in tumour growth, metabolism and hypoxia.

## Material and Methods

### Cell Lines and Culture Conditions

In this study, two HNSCC cell lines UT-SCC-14 (tongue) and UT-SCC-2 (floor of mouth) from the University of Turku were used. These cell lines were cultured in Dulbecco’s modified Eagle’s medium supplemented with 2 mM glutamine, 1% non-essential amino acids, 100 IU/ml penicillin-G, 50 µg/ml streptomycin, and 10% foetal bovine serum (all from GIBCO, Paisly, UK). The cells were given fresh culture media twice per week and were subcultured at confluence after detaching the cells with 0.25% trypsin + 0.02% ethylenediaminetetraacetic acid at a weekly split ratio of 1:4. Cultures in passages 15–25 were used in all experiments. Cells were screened periodically for mycoplasma contamination using DAPI staining and/or the Universal Mycoplasma Detection Kit (ATCC, Manassas, VA, USA).

### Xenograft Mouse Model and Assessment of Tumorigenicity

20 BALB/c (nu/nu) female nude mice were injected subcutaneously in both flanks with 5 × 10^6^ cells suspended in 0.2 ml of phosphate-buffered saline (PBS) using ethical committee-approved standard protocols and procedures. All procedures were performed at the Linköping University’s animal facility and were approved by the local Animal Use and Care Committee (Dnr 126-10). Ten mice were injected with UT-SCC-14 and the remaining ten with UT-SCC-2. The mice were examined twice a week for development of tumours. Within each group of ten animals five received three intraperitoneal injections of cetuximab (Erbitux^®^; 1 mg/injection; Merck KGaA, Darmstadt, Germany) at day 10, 13, and 16 after injection of tumour cells. The size of the tumours was recorded at an interval of 2–3 days. Tumour-bearing mice were sacrificed at day 21, and the tumour tissue was excised, fixed in formalin, and embedded in paraffin. The experiment was repeated twice.

### Tissue Microarray (TMA)

A TMA was constructed from formalin-fixed paraffin embedded tumours at the Swedish Science for Life Laboratory (SciLifeLab) facilities at the Department of Immunology, Genetics, and Pathology at the Rudbeck Laboratory of Uppsala University (Sweden), as previously described [29]. In brief, two 1.0-mm diameter cores from each donor block (duplicate samples) were obtained and assembled in an array format in a recipient TMA block using TMArrayer™ (Pathology Devices, Westminster, MD, USA).

### Immunohistochemistry (IHC)

IHC and slide scanning were performed at the Swedish SciLifeLab facilities in accordance with protocols described elsewhere [29]. In brief, 4-μm TMA sections collected on SuperFrost Plus slides were deparaffinised in xylene, re-hydrated in graded alcohols, blocked for endogenous peroxidase and subjected to heat-induced antigen retrieval. Automated IHC was performed using a LabVision Autostainer 480 S (Thermo Fisher Scientific, Runcorn, UK). Primary antibodies: Ki67 (M7240, Dako, 1:200), EGFR (28–005, Zymed, 1:40), pEGFR (ab32578, Abcam, 1:50), HIF-1α (NB100-131, Novus Biologicals, Littleton, Colorado, USA, 1:8000), GLUT1 (HPRK3890278, Uppsala ak 58494 1:50), CAIV (NB100-131, Novus Biologicals, 1:400), MCT4 (Sc-50329, Santa Cruz Biotechnology, Santa Cruz, CA, USA, 1:400) and MCT1(Sc-365501, Santa Cruz Biotechnology, 1:400) were diluted in UltraAb Diluent (Thermo Fisher Scientific, Fremont, CA, USA), and applied to the slides for 30 min at room temperature. The slides were further incubated with the secondary reagent (anti-rabbit/mouse horse radish peroxidase-conjugated UltraVision; Thermo Fisher Scientific, Runcorn, UK) for 30 min at room temperature. Following the washing steps, the slides were developed for 10 min using the avidin-biotin peroxidase staining technique (Vector Elite; Vector Laboratories, Burlingame, CA, USA) using 3,3-diaminobenzidine as the substrate. The slides were counterstained with Mayer’s hematoxylin for 5 min (Sigma-Aldrich, St. Louis, MO, USA) and coverslipped with Pertex (Histolab AB, Gothenburg, Sweden).

### Slide Scanning and IHC Scoring

To obtain high-resolution digital images, stained IHC slides were scanned with a 20× objective using an Aperio Scan Scope XT Slide Scanner (Aperio Technologies, Vista, CA, USA). Scoring was performed blinded without knowledge of cell line or treatment by two investigators. A definite consensus score was determined by mutual agreement in a separate session. Staining for EGFR, pEGFR, MTC1, MTC4, CAIV and GLUT1 was predominantly membranous and was scored according to the percentage of positively stained cells and staining intensity (<10% = 0, >10% of the cells and weak staining = 1, >10% of the cells and moderate staining = 2, or >10% of the cells and strong staining = 3). Ki67 was used as a proliferation marker and scored as follows: 0–10% = 1, 11–50% = 2, and >50% = 3).

### EPR Probes

LiPc (Clin-EPR, NH, USA) crystals/aggregates were loaded into 23 G needles and compressed to create LiPc oximetry probes using a technique described previously [26–28]. Each probe (approximate inner diameter 400 µm, length approximately 1.4 mm and weight 20–30 µg) was then injected into each tumour with the help of a custom made syringe by inserting and orienting the tip of the needle into the central core of each tumour and pushing the plunger forward propelling the probe into the solid tumour mass.

### EPR Measurements and Analysis

EPR measurements were performed 2–3 days after injection of the LiPc probes using a Bruker Elexsys E540 L band EPR spectrometer equipped with an E540 R36 L band resonator (36 mm sample access), an E540 GCL Triple axis coil set (gradient field strength up to 40 G/cm) and an EPR 066L-AMC L band Microwave Bridge. Mice were anaesthetised by an injection of mixture of ketamine and xylazine as per body weight and placed inside the resonator. EPR spectrometer parameters were: 36 mW applied microwave power, 0.1 G modulation amplitude, time constant 20.48 ms, 10 s sweep time (512 measurement points), centre field approximately 384 G with 3 G sweep width, similar to the settings used in earlier works [27, 28]. Due to the low Q-factor of the resonator, a relatively high microwave power could be applied without excessive saturation. Twenty sweeps were added together for each measurement to a total measurement time of approximately 3 min 20 s. The two probes in each mouse (one in each tumour) were separated by a gradient of 1 G/cm along B_0_. No EPR signal could be detected for the empty resonator.

The recorded EPR spectra were imported into Matlab using the Easyspin toolbox [30] and analysed using in-house developed software. The number and approximate positions of the peaks in each spectrum were determined automatically after filtration by the “rcfilt” function implemented in Easyspin [30]. An interactively adjusted threshold value was applied in the Matlab function “findpeaks”. EPR line widths were then calculated after the peak positions had been refined by parabolic fits to the experimental spectra. Supplementary information, including a brief manual, Matlab code and an example run is published online [31].

## Results

### In vivo Cetuximab Sensitivity

**Fig. 1:**
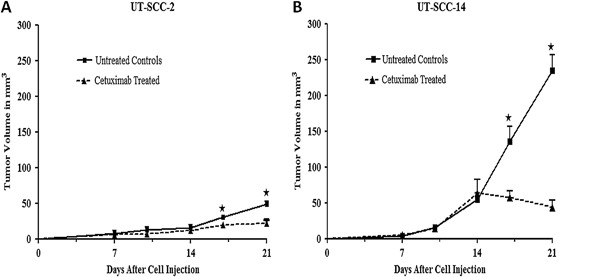
Tumour growth. Xenografts were established in female nude mice (BALB c[nu/nu]) by subcutaneous injection of head and neck squamous cell carcinoma cell lines **a** UT-SCC-14 and **b** UT-SCC-2. Cetuximab (1 mg/injection) was administered by intraperitoneal injection at day 10, 13 and 16. The tumour size was recorded at an interval of 2–3 days, *n* = 10-14. Letter **a** indicates that the tumour volume of untreated controls is significantly different than that of cetuximab-treated group (*P* < 0.05)

We have previously screened a large number of HNSCC cell lines to determine their cetuximab sensitivity in vitro [29]. For this study two cell lines, UT-SCC-14 with intrinsic cetuximab sensitivity (ICmabS) of 0.15 and UT-SCC-2 with an ICmabS of 0.96 were chosen. The cells were injected subcutaneously in the flanks of female nude mice and all injections gave rise to tumours. From day 7 the tumour volume was measured and at day 10, 13 and 16 treated mice received cetuximab. In both cell lines (UT-SCC-2 and UT-SCC-14) cetuximab had effect on the tumour volume but the effect was more pronounced on UT-SCC-14 xenografts (Fig. 1).

### Tumour Oxygenation

**Fig. 2:**
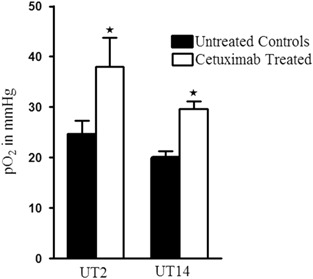
Partial oxygen pressure in response to cetuximab treatment. Partial oxygen pressure in response to cetuximab treatment analysed by EPR measurements in untreated controls and cetuximab-treated UT-SCC-2 (UT2) and UT-SCC-14 (UT14) xenografts at day 21. *Asterisk* shows that the partial oxygen pressure values of untreated controls are significantly lower compared to the cetuximab-treated group (*P* < 0.05) tested with one way ANOVA and post hoc Tukey’s HSD test, *n* = 10–14

Tumour oxygenation for the xenografts was measured using EPR oximetry at day 21 and an oxygenation approximately 50% higher was measured in cetuximab-treated tumours in both cell lines compared to untreated controls (Fig. 2). The possible relationship between the tumour volume and the partial oxygen pressure was analysed using the Pearson correlation test. No correlation between these parameters in UT-SCC-2 xenografts (*R* = −0.13, *P* = 0.59) could be found, but in UT-SCC-14 xenografts a very weak correlation was observed (*R* = −0.3, *P* = 0.05).

### Expression of Proteins Involved in Proliferation

A TMA was constructed from untreated and cetuximab-treated UT-SCC-14 and UT-SCC-2 xenografts, and the expression of EGFR, pEGFR and Ki67 was determined by IHC.

**Fig. 3:**
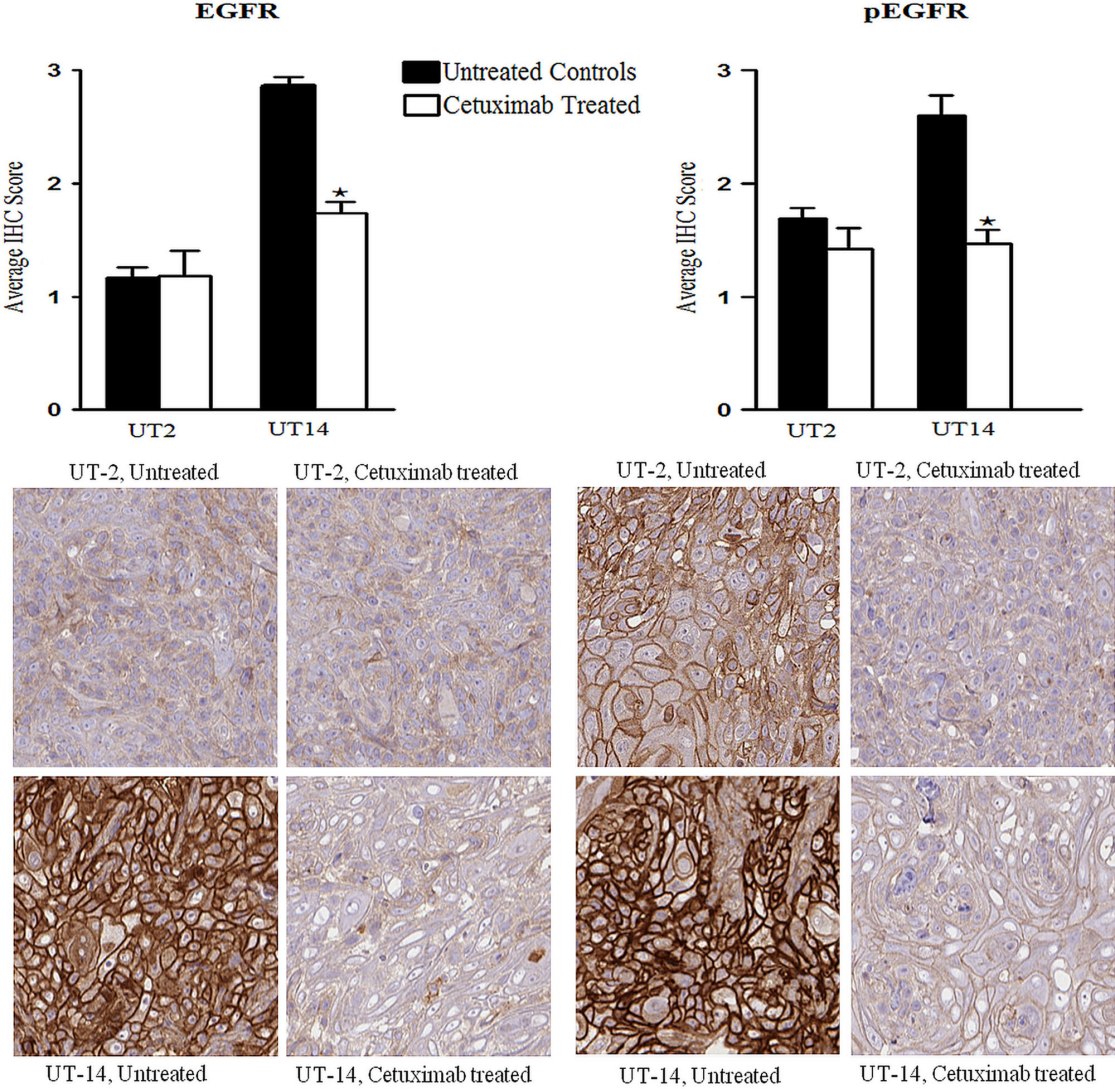
EGFR and pEGFR protein expression. Xenografts were established in female nude mice (BALB c[nu/nu]) by subcutaneous injection of head and neck squamous cell carcinoma cell lines UT-SCC-2 (UT-2) and UT-SCC-14 (UT-14). Cetuximab (1 mg/injection) or PBS was administered by intraperitoneal injection at day 10, 13, and 16. A tissue microarray was constructed from tumours harvested at day 21, and the expression of epidermal growth factor receptor (EGFR) and active phosphorylated EGFR (pEGFR) was evaluated by immunohistochemistry (IHC) in untreated controls and cetuximab-treated tumour specimens. Bar graphs showing the IHC staining score for EGFR and pEGFR, and one way ANOVA and post hoc Tukey’s HSD test were used to test differences between treated and untreated groups (*p ≤ 0.05), *n* = 10–14

Untreated UT-SCC-14 xenografts showed a higher level of EGFR, pEGFR and Ki67 expression as compared to untreated UT-SCC-2 xenografts (Fig. 3). After 9 days of cetuximab treatment the expression of EGFR, pEGFR and Ki67 was significantly decreased in UT-SCC-14 tumours as compared to untreated controls. In UT-SCC-2 xenografts, on the other hand, there were no significant changes in the EGFR, pEGFR or Ki67 expression after cetuximab treatment (Fig. 3).

### Expression of Proteins Involved in Metabolism and Hypoxia

The expression of three proteins known to be involved in tumour metabolism, monocarboxylate transporter 1 (MCT1), monocarboxylate transporter 4 (MCT4) and glucose transporter 1 (GLUT1) was determined by IHC.

**Fig. 4:**
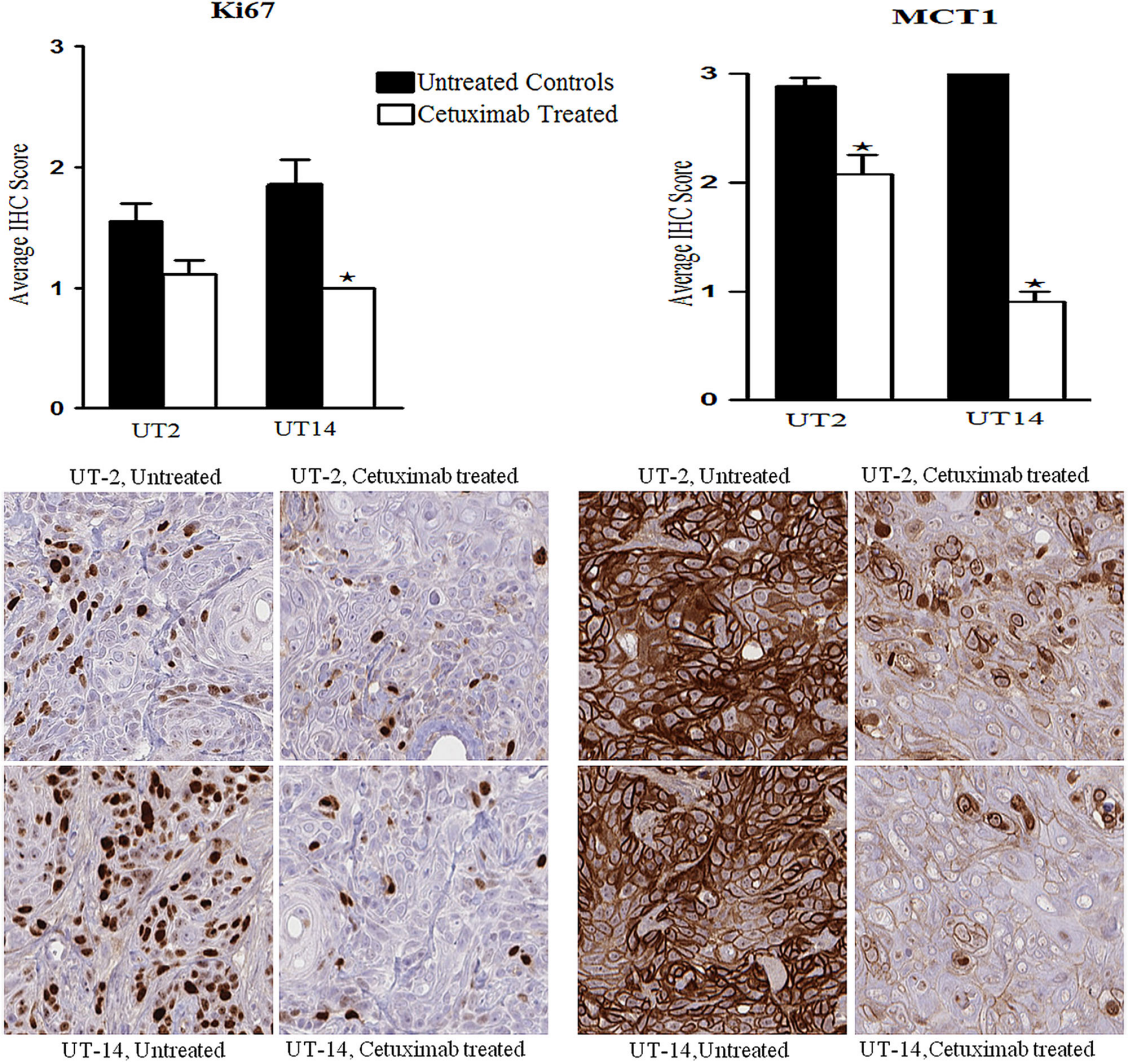
Ki67 and MCT1 protein expression. Xenografts were established in female nude mice (BALB c[nu/nu]) by subcutaneous injection of head and neck squamous cell carcinoma cell lines UT-SCC-2 (UT-2) and UT-SCC-14 (UT-14). Cetuximab (1 mg/injection) or PBS was administered by intraperitoneal injection at day 10, 14, and 17. A tissue microarray was constructed from tumours harvested at day 21, and the expression Ki67 (proliferation marker) and MCT1 was evaluated by immunohistochemistry (IHC) in untreated controls and cetuximab-treated tumour specimens. Bar graphs showing the IHC staining score for Ki67 and MCT1, and one way ANOVA and post hoc Tukey’s HSD test were used to test differences between treated and untreated groups (*p ≤ 0.05), *n* = 10–14

**Fig. 5:**
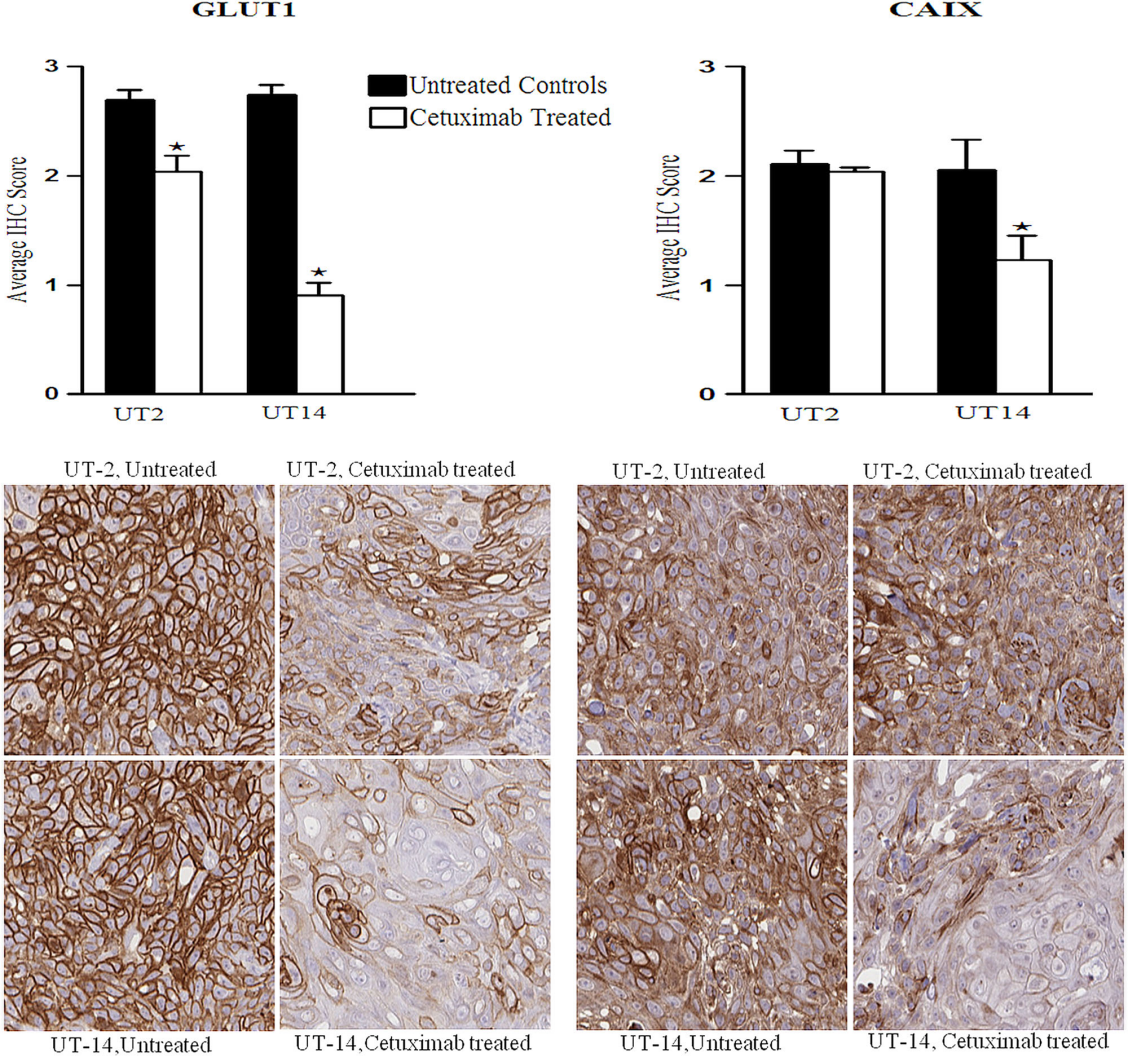
GLUT1 and CAIX expression. Xenografts were established in female nude mice (BALB c[nu/nu]) by subcutaneous injection of head and neck squamous cell carcinoma cell lines UT-SCC-2 (UT-2) and UT-SCC-14 (UT-14). Cetuximab (1 mg/injection) or PBS was administered by intraperitoneal injection at day 10, 14, and 17. A tissue microarray was constructed from tumours harvested at day 21, and the expression of GLUT 1 and CAIX was evaluated by immunohistochemistry (IHC) in untreated controls and cetuximab-treated tumour specimens. Bar graphs showing the IHC staining score for GLUT1 and CAIX, and one way ANOVA and post hoc Tukey’s HSD test were used to test differences between treated and untreated groups (*p ≤ 0.05), *n* = 10–14

The MCT1 and GLUT1 expression was significantly decreased in the cetuximab-treated groups of both cell lines but differences were more pronounced in UT-SCC-14 xenografts (Figs. 4,5). Interestingly, we did not find any differences in MCT4 expressions between untreated and cetuximab-treated xenografts of both UT-SCC-2 and UT-SCC-14 (data not shown).

The protein expression of hypoxia inducible factor 1α (HIF1α) and carbonic anhydrase IX (CAIX), two proteins known to be involved in hypoxia, was also investigated.

**Fig. 6:**
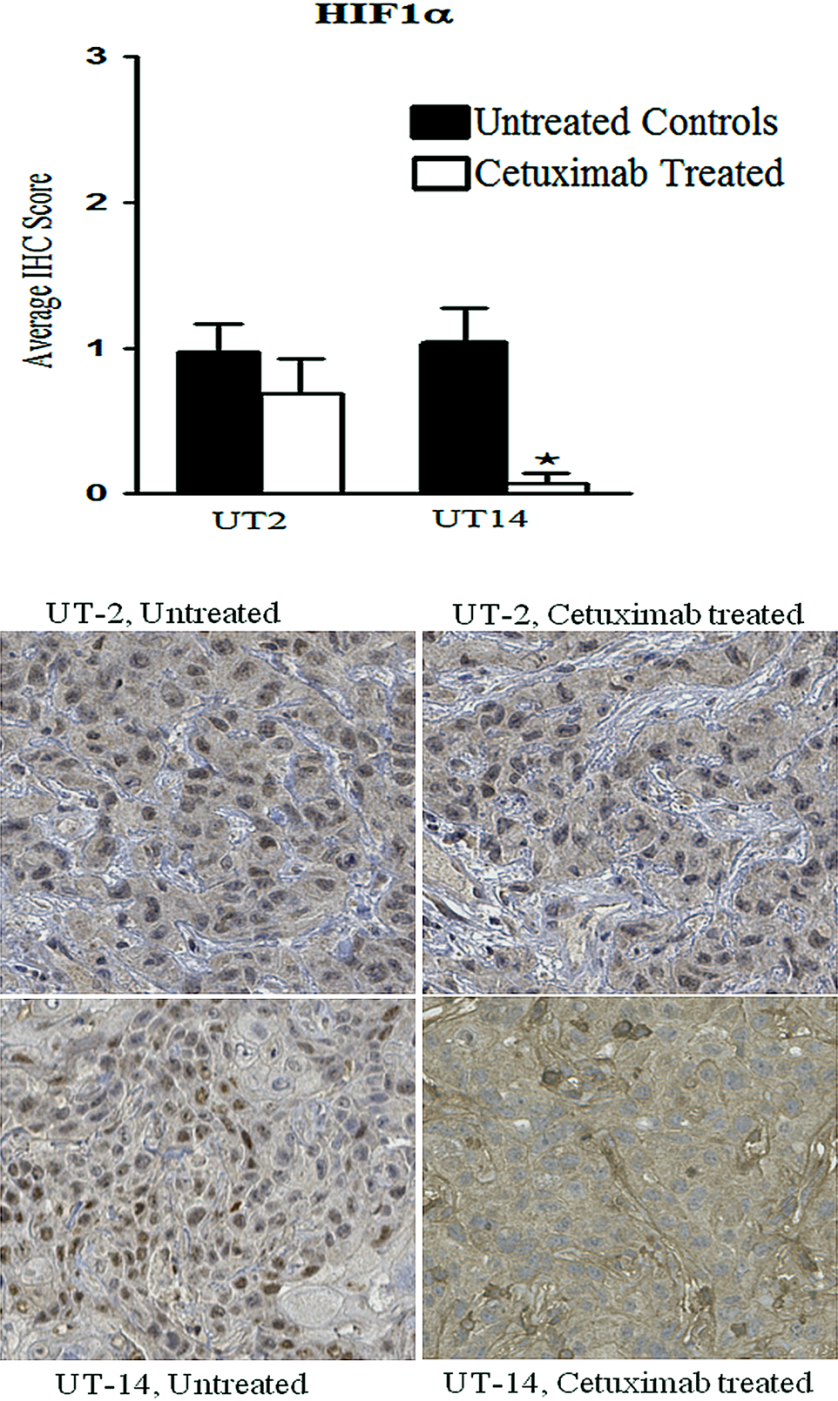
Nuclear HIF-1α protein expression. Xenografts were established in female nude mice (BALB c[nu/nu]) by subcutaneous injection of head and neck squamous cell carcinoma cell lines UT-SCC-2 (UT-2) and UT-SCC-14 (UT-14). Cetuximab (1 mg/injection) or PBS was administered by intraperitoneal injection at day 10, 14, and 17. A tissue microarray was constructed from tumours harvested at day 21, and the expression of nuclear HIF-1α was evaluated by immunohistochemistry (IHC) in untreated controls and cetuximab-treated tumour specimens. Bar graphs showing the IHC staining score for HIF-1α. One way ANOVA and post hoc Tukey’s HSD test were used to test differences between treated and untreated groups (*p ≤ 0.05), *n* = 10–14

The nuclear expression of HIF-1α was decreased in UT-SCC-14 tumours after cetuximab treatment but was mostly unchanged in tumours from the UT-SCC-2 (Fig. 6). However, the overall non-nuclear HIF-1α expression did not show any significant differences between the groups (data not shown).

The CAIX expression was significantly reduced in cetuximab-treated UT-SCC-14 xenografts when compared to the untreated controls but in UT-SCC-2 xenografts cetuximab treatment did not change the expression of this protein (Fig. 5).

## Discussion

Oxygen is a key regulator and stimulant for cell growth and metabolism-related processes, and aerobic environment is important in maintaining healthy cell activities. Tumour development might however alter to a large extent the oxygenation status due to the poor vasculature of the architectural network and the environmental gradients around the blood vessels [32]. In this study, tumour oxygen pressure was measured using EPR oximetry in the tumours generated by both the HNSCC cell lines to evaluate the fate of oxygen in the tumour mass and to get a clear understanding of the correlation between oxygenation in a well-developed tumour mass and in a cetuximab-treated tumour mass.

Our method of choice for EPR oximetry was to use the particulate LiPc probes [26–28], which are known to be robust, sensitive and minimally invasive after the initial insertion of the probes in the tumours. The in vivo EPR spectra obtained in this study had in some cases poor signal-to-noise ratio (SNR) and the spectrometer parameters used for the measurements were therefore a trade-off between maximum spectral resolution (as low modulation amplitude as possible), minimal microwave power to avoid excessive saturation broadening and the SNR needed to determine oxygen tension with sufficient accuracy and precision. Broadening caused by microwave saturation may be less serious in spite of the relatively high microwave power used since the amplitude of the microwave field B1 is considerably reduced by the low Q-value in comparison with a conventional cavity at the same incident microwave power. Due to the much lower sensitivity than of conventional spectrometers the signals had to be digitally filtered before the pO_2_ values were evaluated. Digital filtration may cause additional broadening of up to 10% [31]. Overmodulation has been employed to improve the SNR and the true linewidth was obtained by post treatment of the data as described by Robinson, Mailer and Reese [33]. The in-house developed software was used to optimise measurement precision in oxygen pressure measurements by applying signal smoothing and subsequent refinement of the peak-to-peak line width by parabolic fitting to the experimental spectrum rather than directly measuring the width of a noisy line [31].

Tumour oxygenation has a spatial heterogeneity in HNSCCs [34] that might influence the measurement process. Thus, the EPR oximetry probes used in the present study (approximate diameter 400 µm and length 1.4 mm) extend over several tumour intervascular distances and subsequently report an average oxygen pressure for this area similar to polarographic electrodes [35]. In this respect they have a similar behaviour to that of Oxylite fluorescence probes as shown by Elas and colleagues [25] with a direct comparison of measurements in FSa fibrosarcomas grown in the legs of C3H mice. The extent to which the additional temporal oxygenation heterogeneity due to perfusion fluctuations in the tumour capillaries [12] influences the measurements is considered smaller as individual EPR measurements lasted on average about 3 min. Nevertheless, these sources of heterogeneity might explain to a certain extent the difficulties in finding a clearer correlation between tumour size and oxygenation in the given population of animals.

To improve the cure rates and expedite recovery of head and neck patients, it is important to identify reliable predictive biomarkers. Here, we investigated the tumour oxygenation and the expression of proteins involved in proliferation, metabolism and hypoxia in untreated and cetuximab-treated xenografts. In this study one cetuximab-sensitive cell line (UT-SCC-14, established from a tongue tumour) with a high EGFR expression and one resistant cell line (UT-SCC-2, established from a floor of mouth tumour) with a low EGFR expression were used. Both were known to form xenografts in mice [36] and to harbour wild-type *KRAS* and *BRAF* (unpublished data). The UT-SCC-14 cell line, which showed a high sensitivity to cetuximab, exhibited a very high expression of both total and activated EGFR whereas the UT-SCC-2 cell line, which is less sensitive, had lower levels of EGFR. We and others have previously demonstrated that there is no correlation between the level of EGFR expression and cetuximab sensitivity [36–38].

In this study we investigated the influence of cetuximab on the EGFR expression and activation in vivo using IHC. In the cetuximab-sensitive xenografts investigated parameters (EGFR, pEGFR, and Ki67) were significantly decreased and in the less sensitive tumours no significant differences were noticed. These data are in line with observations from one of our recent in vitro studies in which three tongue cancer cell lines (LK0412, LK0824 and LK0902) were investigated. We found that cetuximab treatment resulted in a reduction in the EGFR and pEGFR expression in the most sensitive cell line LK0902. In contrast, cetuximab treatment had no effect on the EGFR and pEGFR expression of LK0824 cells, which exhibited an intermediate sensitivity to cetuximab, whereas an increase in EGFR and pEGFR was observed in the resistant cell line LK0412.

We also investigated the expression of proteins involved in metabolism and hypoxia. The transporter MCT1 is known to be an importer of L-lactate and ketone bodies into cells. MCT1 has been proposed as a possible metabolic therapeutic target in HNSCC since an increased expression has been found in the most metabolic and proliferative active HNSCC cells [39]. In this study, a high expression of MCT1 was detected in xenografts from both cell lines probably due to a high metabolic and proliferative activity in these xenografts. Cetuximab treatment reduced the MCT1 expression most likely due to the detected decrease in proliferation (Ki67 staining), and increased oxygenation (EPR oximetry). The transporter MCT4 is a marker of glycolysis, lactate and ketone body release, and has been shown to be a direct transcription target of HIF-1α and markedly upregulated in hypoxia [40]. In the current study, while MCT4 expression was high in the tumours as expected, the expression levels did not alter significantly after the cetuximab treatment. This might be due to that the detected downregulation of MCT1 is enough for regulation of the lactate transport after reduction of tumour mass and increased oxygenation.

The HIF-1α expression was seen to be accumulated in the tumour mass specifically in the UT-SCC-14 xenografts, which might be due to the hypoxic condition present in the tumour mass. Cetuximab treatment showed reduction in the nuclear accumulation of HIF-1α, while the overall HIF-1α expression was not significantly altered. In line with these results, Li et al have shown in HNSCC cell lines that cetuximab can downregulate HIF-1α by inhibiting the transcriptional activity [40]. More recently, Li and coworkers reported that Cetuximab reverses the Warburg effect in cancer cells via inhibiting HIF-1-regulated lactate dehydrogenase A [41]. These results together with our results in this study indicate a connection between HIF-1α and hypoxia with the EGFR pathway where proteins like Ras, Akt and mTOR could be central.

CAIX is a transmembrane protein and is a tumour-associated isoenzyme. It is regulated by HIF-1α, overexpressed in the hypoxic tumours and is involved in cell proliferation [42]. CAIX is a biomarker of cellular hypoxia and in this study the CAIX expression was high in both UT-SCC-2 and UT-SCC-14 xenografts. However, after cetuximab treatment a downregulation of CAIX was only found in UT-SCC-14 xenografts, which may be due to the significant downregulation of HIF-1α detected in these xenografts.

GLUT1 facilitates the cellular uptake of glucose through the plasma membrane and it is overexpressed in most of tumours including HNSCC. In the present study, the GLUT1 expression was high in both UT-SCC-2 and UT-SCC-14 xenografts. Interestingly, after cetuximab treatment a decreased expression was found in both tumours but the decrease was more pronounced in UT-SCC-14. This result together with all our results in this study show a potential connection between increased oxygenation and decreases in the hypoxia dependent proteins GLUT1, MCT1, CAIX, HIF-1α (nuclear) and in Ki67 expression in HNSCC xenografts.

Taken together, our results show that cetuximab treatment affects the tumour growth and the tumour partial oxygen pressure as measured by LiPc EPR oximetry. Furthermore, we found a possible connection between the partial oxygen pressure of the tumours and the expression of proteins involved in tumour growth, metabolism and hypoxia.

## Electronic supplementary material

Supplementary Information
