# Supplementary material for: EPR Oximetry of Cetuximab-Treated Head-and-Neck Tumours in a Mouse Model
Source: Cell Biochem Biophys. 2017 Jul 29;75(3):299–309. doi: 10.1007/s12013-017-0814-5 (PMC5691101; doi:10.1007/s12013-017-0814-5)
Supplement: Supplementary file 1 — Supplementary Information [file 12013_2017_814_MOESM1_ESM.pdf]

# Software for *in vivo* oximetry by EPR imaging

Håkan Gustafsson, Department of Radiology Norrköping and Department of Medical and Health Sciences (IMH), Linköping University, Linköping, Sweden
E-mail: hakan.l.gustafsson@liu.se
Anders Lund, Department of Physics, Chemistry and Biology, IFM, Linköping University, SE-581 83 Linköping, Sweden
E-mail: anders.lund@liu.se

Abstract: *In vivo* measurements were performed to obtain the oxygen pressure ($pO_2$) in tumour tissue of mice using EPR imaging (EPRI). Lithium phthalocyanine (LiPc) oximetry probes were used. The signals of two probes were separated by a field gradient along the static field of a Bruker Elexsys E540 L band EPR spectrometer. The oxygen dependence of the EPR line width was utilized to measure the $pO_2$ values. Software developed in Matlab was employed to automatically compute the line widths and $pO_2$ values from the digitally recorded spectra. The original software has been modified to allow post-processing of the experimental data using filter functions available in Easyspin. The accuracy of the measured line widths and $pO_2$ values is discussed.

## 1. Introduction

Several methods have been developed in the past to quantify tumour oxygenation for applications in radiation therapy [1]. Procedures relying on electrochemical and enzymatic reactions have been employed for measuring tumour hypoxia [2,3]. Physical measurements have also been used [4]. Electron paramagnetic resonance (EPR) oximetry has recently been proposed as an alternative method for the determination of oxygen concentration *in vivo* [5-8]. EPR oximetry is based on the observation that the EPR linewidths of certain paramagnetic species are broadened in the presence of molecular oxygen, which itself is paramagnetic [9]. The partial pressure of oxygen can be measured non-invasively *in vivo* by means of the EPR line width of a paramagnetic probe present in the tissue. EPR oximetry is currently evolving towards clinical use both in the form of low frequency *in vivo* EPR spectroscopy and *in vivo* imaging (Electron Paramagnetic Resonance Imaging, EPRI). Quantitative measurements and/or images of the oxygen partial pressure in the tissue of interest have been obtained. Initial clinical applications have been reported [7].
Lithium phthalocyanine (LiPc) probes are robust, sensitive and minimally invasive. The EPR spectra in initial studies [8] had, however, a poor signal to noise ratio (SNR). The spectrometer parameters were therefore a trade-off between spectral resolution and sufficient SNR to determine the oxygen concentration. Overmodulation has been employed to improve the SNR, and the true linewidth was obtained by post-treatment of the data using methods described in [10]. An adaptive filter method has also been developed to improve the SNR [11]. These methods require special software or longer measurement times, however. Freely available filter functions [12] and tools in Matlab were applied for the post-processing of the signals in the present work. The obtained peak-to-peak line widths were subsequently refined by parabolic fits to the experimental spectra. The software was applied in an ongoing study [13] to investigate the effect of cetuximab treatment on tumour growth. In this work the original Matlab program [14] has been updated and a version employing signal smoothing with moving average filters has been developed. The performance of the software has been investigated. The spectral resolution obtained with this software, was increased in comparison with the original one.

## 2. EPR measurements

Lithium phthalocyanine (LiPc) (Clin-EPR, NH, USA) crystals/aggregates were loaded into 23 G needles and compressed to obtain LiPc oximetry probes as described by Khan et al [15]. Two probes were usually employed in each EPR measurement. EPR measurements were performed using a Bruker Elexsys E540 L band EPR spectrometer equipped with an E540 R36 L band Resonator (36 mm sample access), an E540 GCL Triple axis coil set (gradient field strength up to 40 G/cm) and an EPR 066L-AMC L band Microwave Bridge. Typical EPR spectrometer parameters were: microwave

power 36 mW, modulation amplitude 0.1 G, time constant 20.48 ms, sweep time 10 s (512 measurement points), centre field (B0) approximately 384 G, sweep width 3 G. 20 sweeps were added, yielding a total measurement time of approximately 200 s. The two probes were separated by a gradient of 1 G/cm along B0.

### 3. EPR analysis

The recorded EPR spectra were imported into Matlab employing the Easyspin toolbox [12] and analysed using in-house developed software. The signal obtained after applying the "rcfilt" or "datasmooth" functions implemented in Easyspin was analysed to obtain the number and positions of the peaks in each spectrum. An automatic procedure was applied using the Matlab function "findpeaks" to detect lines with an amplitude exceeding an adjustable threshold value. EPR line widths were calculated after the peak positions had been refined by parabolic fits to the experimental spectra. The obtained linewidths and amplitudes were used to display theoretical spectra, with Gaussian, Lorentzian or Voigt shapes [16], together with the experimental ones. The line width of the EPR signal was converted to $pO_2$ (mm Hg) by calibration measurements at different partial oxygen pressures as described in [15].

**TABLE 1**. Oxygen pressures obtained from the linewidths of two LiPc probes

| [a)]Filter/setting | **Probe I/Low-field line** | | **Probe II/High-field line** | |
|---|---|---|---|---|
| | **Width (G)** | **$pO_2$ (mm Hg)** | **Width (G)** | **$pO_2$ (mm Hg)** |
| **[b)]Flat/m=5** | 0.215 | 33.3 | 0.161 | 23.5 |
| **[b)]Binom/m=5** | 0.221 | 34.4 | 0.159 | 23.2 |
| **[b)]SavGol/m=5** | 0.215 | 33.4 | 0.159 | 23.1 |
| **[c)]RC/Tc=5 ms** | 0.216 | 33.6 | 0.177 | 26.5 |
| **[d)] unfiltered** | 0.201 | 30.7 | 0.161 | 23.5 |

[a)]Filter functions implemented in Easyspin. [b)]An average over 2m+1 = 11 data points was made. [c)]The signal was filtered with a time constant Tc = 5 ms by an RC-filter**.** [d)]Post-processing was not applied.

The results in Table 1, obtained from the analysis of data obtained in an in vivo study [13], indicate that the $pO_2$ values may differ with up to ±10% depending on the filter type at fixed settings. A value of Tc = 5 ms was employed to conform with the conditions in [13], while averaging over 11 data points (m=5) was used for the three types (b) of moving average smoothings. The width obtained from the unfiltered signal (d) may be difficult to estimate accurately due to the low SNR. Probe II has a narrow linewidth, which might become broadened by the RC filter as indicated in the Table. This possibility was investigated by applying a number of filter settings, Fig.1. The results indicate that the $pO_2$ values obtained in initial studies [13] might have been somewhat overestimated, especially at hypoxic conditions with narrow EPR line widths. The "EPR_width_RC" software may still be applied, by using an option in the input/output dialogue to interactively decrease the Tc value. The SNR may become low, however.
The line widths obtained with the Easyspin functions "savgol" and "binom" were nearly unaffected by the filter settings (m) over a large interval and had a satisfactory SNR at m = 15. The functions have been implemented in EPR_width_RCM" by a slight revision of the original code. The "flat" option was also included, although not employed due to the unsatisfactory performance observed in Fig.1 and in previous works [12(b)].

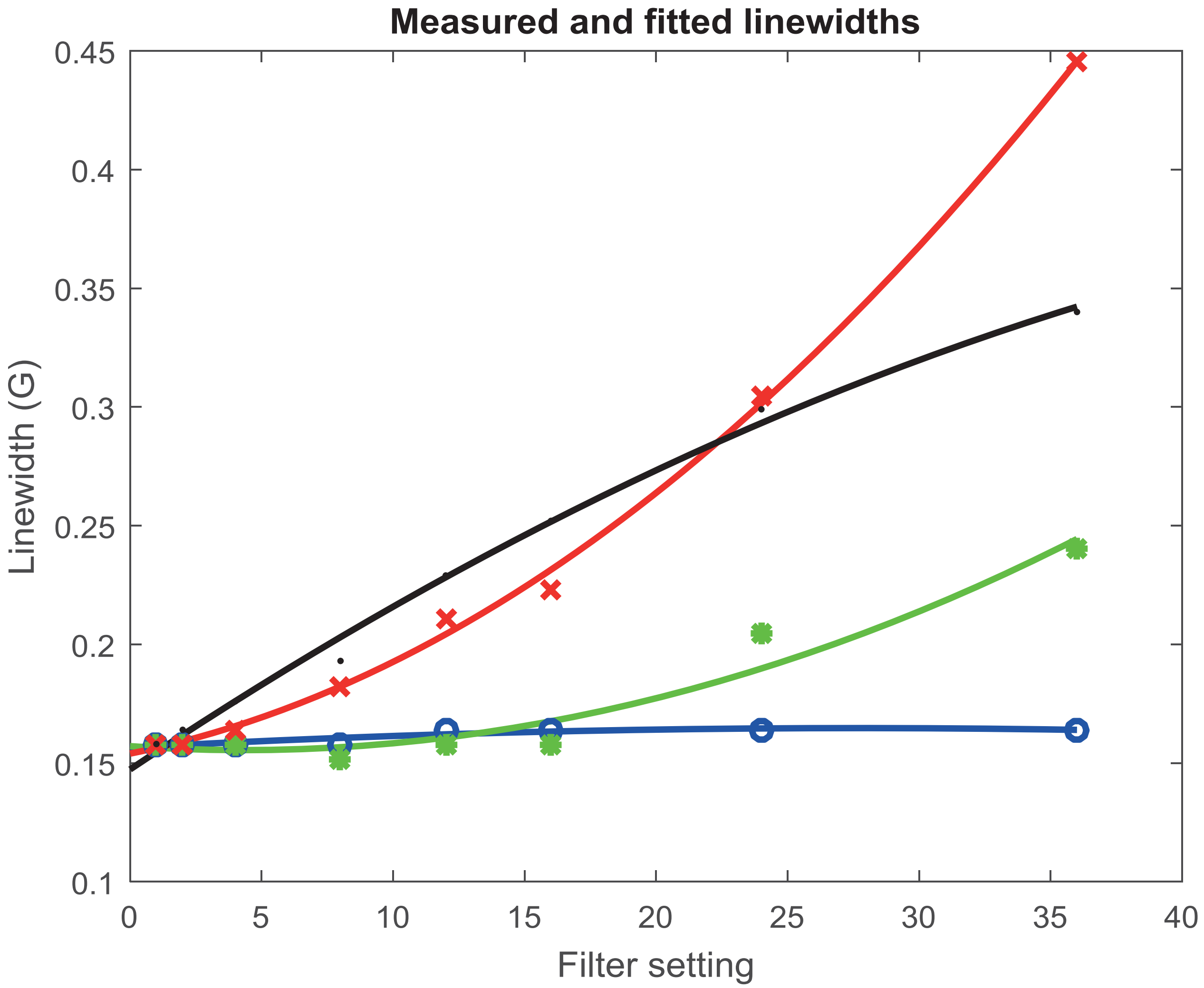


**Fig.1.** Linewidths obtained by applying different filter settings, using moving average smoothing with the ”datasmooth” function in Easyspin. Smoothing was performed over 2m+1 lines, with m = filter setting. Linewidths measured with the ”binary”, ”savgol” and ”flat” options [12(b)] are marked by symbols in blue, green and red. Widths obtained with the ”rcfilt” option, with filter settings in ms units, are in black. The solid lines represent parabolic fits to the measured widths.

## 4. Applications

The initially developed software, ”EPR_width_RC”, has so far been employed in a few studies [8,13]. The $pO_2$ level was determined after applying an RC-filter, typically with a time constant (Tc) of 5 ms. The results obtained in this work demonstrate that a slight broadening of the line widths may then occur, yielding ca 10 % too high $pO_2$ levels. Broadening can be avoided by decreasing Tc, but the SNR may become low. Moving average filter functions were implemented in the ”EPR_width_RCM” software, in an attempt to avoid line broadening. The results indicated that the options ”binom” and ”savgol” available in Easyspin [12(b)] could be used over a wide range of filter settings, with averaging over 2m+1 field points. The measured line widths remained constant up to m = 15, allowing determination of the linewidths at a satisfactory SNR value. Fig.1 indicates that even higher m values might be used with the binomial filter. This software has not yet been used in practical applications, however.

## 5. Input/Output *dialogue using software “*EPR_width_RCM”

The input/output dialogue with ”EPR_width_RCM”, is similar to that of the original program [14] with the difference that the filter type has to be specified. An example is given below.

>> EPR_width_RCM

C:\Users\andlu77\Documents\MATLAB\ESRDOS\t140922m.DTA

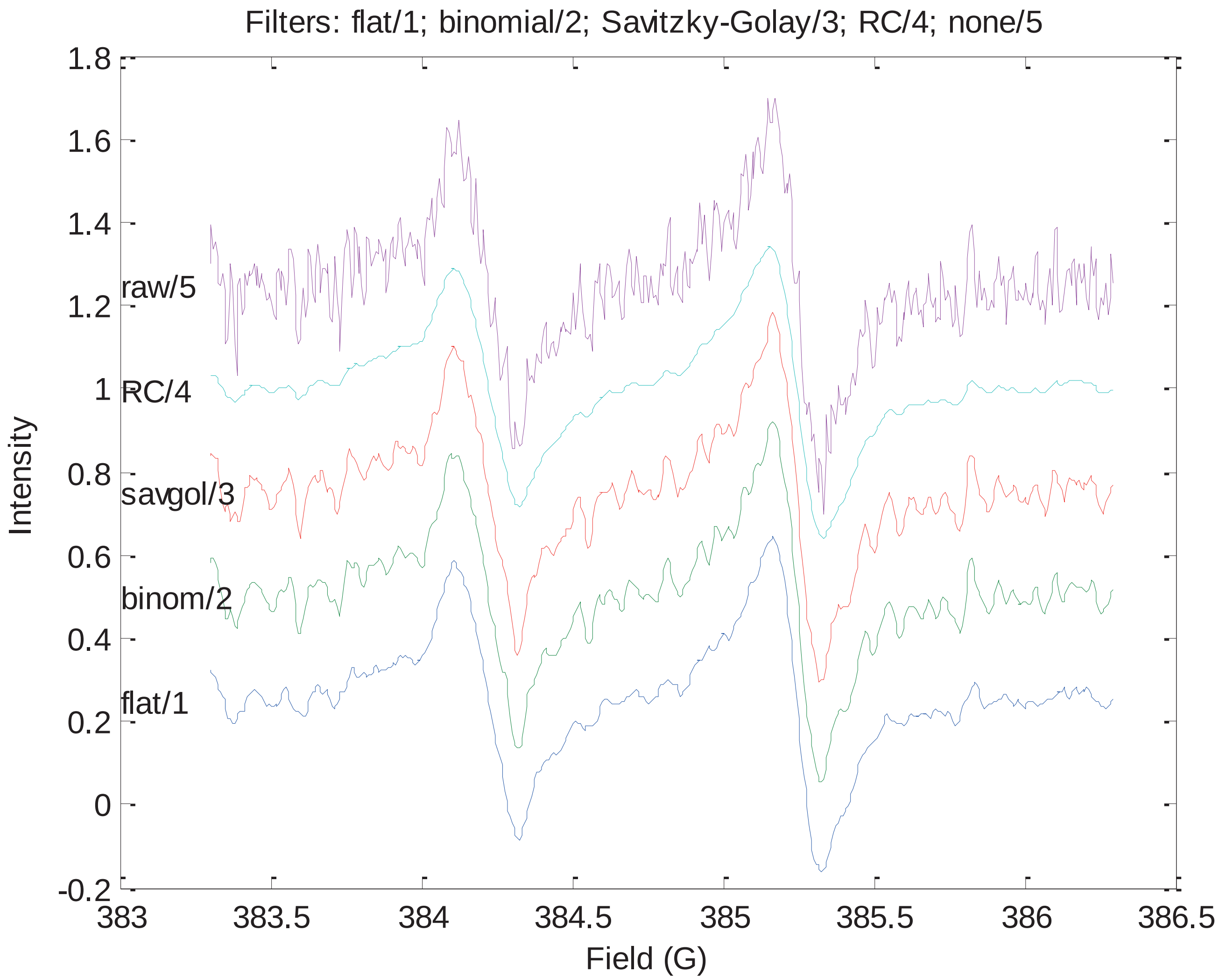


Filters: flat/1; binomial/2; Savitzky-Golay/3; RC/4; none/5:2

Current moving average number=5

Reset moving average number, 0 if OK:15

Filters: flat/1; binomial/2; Savitzky-Golay/3; RC/4; none/5:2

Current moving average number=15

Reset moving average number, 0 if OK:0

Current upper/lower tresholds = 0.30 0.30

Reset upper treshold, (0 if OK):0.35

Reset lower treshold, (0 if OK):0.35

Current upper/lower tresholds = 0.35 0.35

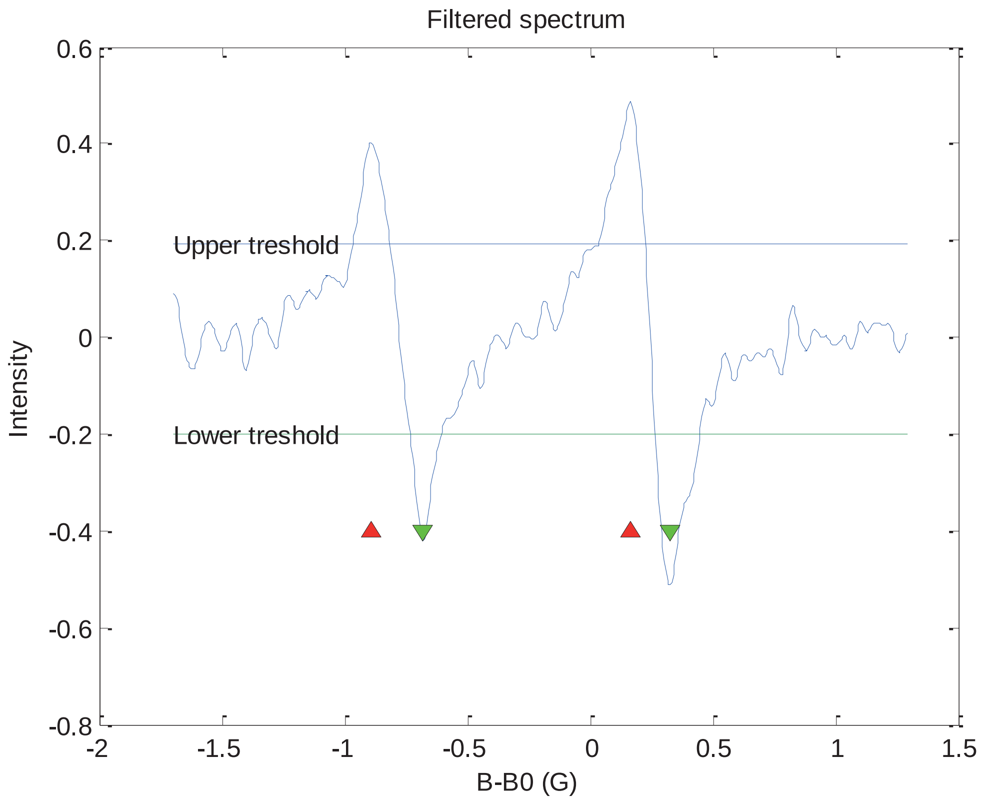


Reset upper treshold,(0 if OK):0
Reset lower treshold,(0 if OK):0

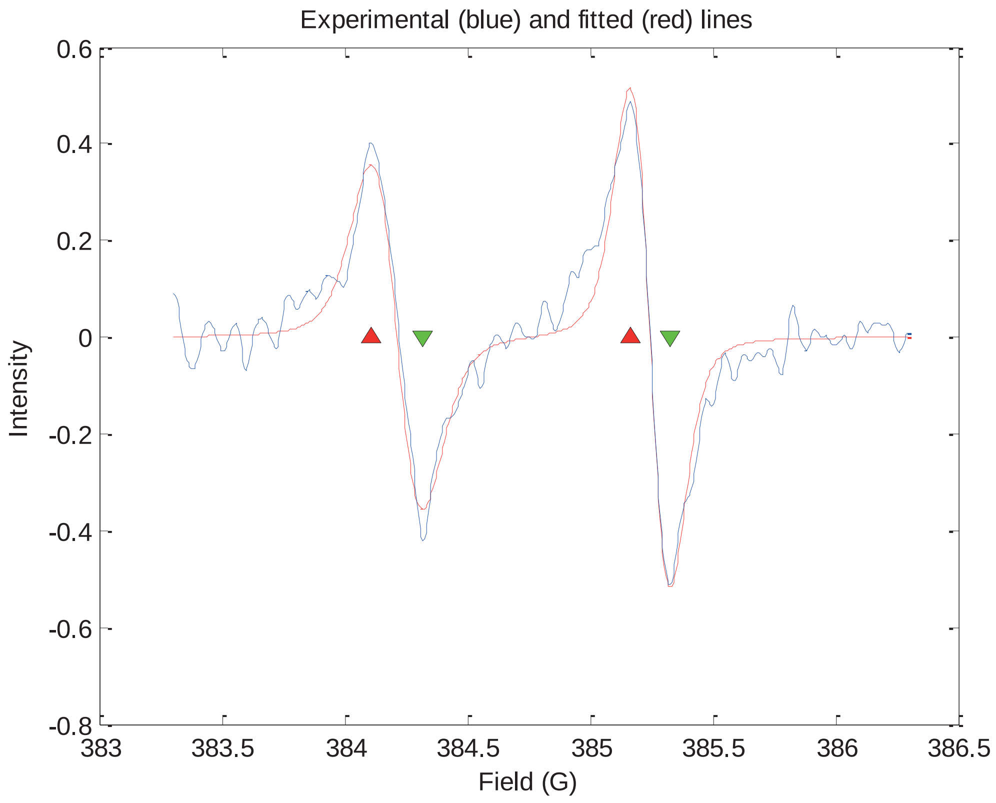


[pO2] = 32.67 +- 0.02 mm Hg, Width(exp.) = 0.211 G, Width(fit) = 0.211 G
[pO2] = 23.94 +- 0.14 mm Hg, Width(exp.) = 0.164 G, Width(fit) = 0.163 G

## 6. Performance

The errors in the $pO_2$ values given in the output are solely attributed to the differences of the line width values, calculated from the distance between the peak marks of each line, and by a fitting procedure. Those errors are usually small. As discussed above, larger errors may occur by unsuitable data processing, e.g. by excessive filtering. Inaccuracies in the calibration of the EPR probes can give rise to errors of the oxygen concentrations that at present are difficult to estimate.

## 7. Software

The software used in earlier applications [8,13] is available at http://www.liu.se/simarc/downloads/?l=en. The folder EPR_oximetry.zip contains a manual, the Matlab code, and example data. Software prepared for this work will be available at the same site, containing the following items in the folder EPR_oximetry_RCM.zip:

- Manual, "EPR_width_RCM.pdf". The analysis of the data is performed interactively by following the instructions on the screen.
- Matlab code, "EPR_width_RCM.m". The software is compatible with Matlab versions up to R2016a.
- Spectrum files: Spectrum and parameter files have the format used in Bruker spectrometers, see [12(b)] for the slight software modifications with data from other instruments.
- Matlab code ""EPR_width_RC_rev.m". The revised code is compatible with Matlab versions up to R2016a.

The results of this work indicate that the moving average filters "binom" and "savgol" implemented in "EPR_width_RCM.m" are well suited for linewidth measurements of narrow EPR signals. As shown in Fig.1 an RC-filter may broaden the EPR lines significantly, while the line width is nearly independent of the settings of the "binom" filter. A satisfactory SNR value was also obtained.

## 8. Operation

Easyspin must be installed, following the procedure in [12(b)]. The data are read by entering the file name, e.g. "t140922m"in "EPR_width_RC.m" or by clicking on "t140922m.DTA" in a list of files that is automatically displayed in "EPR_width_RCM.m", using the code reproduced below. The data formats of other commercial spectrometers may differ, requiring slight modification of the instructions to load the experimental files, see [12(b)]. The analysis of the data is performed interactively by following the instructions on the screen.

## 9. EPR_width_RCM.m

```
function EPR_width_RCM  % Oximetry using LiPc EPR line-width
close all; clear;%clf
[x,y,~,File]=eprload;    % Exp. spectrum from Bruker file
disp(File)               % EasySpin must be installed
nx=size(x,1);
X=x-round(x(1)+x(nx))/2;
splitt=30;               % Minimum separation of peaks in 'findpeaks'
Tc=5;                    % RC time constant(ms)
m=5;                     % 2*m+1 averaged points
L_G=0.5;                 % Lorentz/Gauss line-width ratio
vakt=[0.30 0.30];        % upper/lower signaltresholds
R=(y-mean(y))/(max(y)-min(y));                   % R=unfiltered signal
while Tc*m>0                                     % Select filter
    [A,noisA,val] = Y_filt(x,R,Tc,m);            % A=filtered signal
    figure(2)
    plot(X,A+.15,'r',X,R-mean(R)-.15,'b'); % R=raw, A=filtered signal
    xlabel('B-B0 (G)'); ylabel('Intensity')
    title('Raw (blue) and filtered (red) spectra')
    if val==4
        fprintf('Current time constant=%3.1f ms\n',Tc);
        Tc=input('Reset time constant, 0 if OK:');
    elseif val==5
        fprintf('No filter applied\n');
        Tc=0; m=Tc;
    elseif 1<=val && val<=3
```

```
        fprintf('Current moving average number=%u\n',m);
        m=input('Reset moving average number, 0 if OK:');
    end
end
while max(vakt)>0               % Select treshold levels
    Y=A; noise=noisA;dy=-0.4;
    figure(3);
    plot(X,Y);
    xlabel('B-B0 (G)'); ylabel('Intensity'); title('Filtered spectrum');
    hold on
    min_L=vakt(1)*(max(Y)+noise);  min_R=vakt(2)*(-min(Y)+noise);
    Y_L(1:nx)=min_L;  Y_R(1:nx)=-min_R;
    plot(X,Y_L,X,Y_R);           % upper/lower treshold levels
    text(X(1),min_L,'Upper treshold'); text(X(1),-min_R,'Lower treshold');
    [~,pos_L]=findpeaks(Y,'MINPEAKHEIGHT',min_L,'MINPEAKDISTANCE',splitt);
    NtopL=max(size(pos_L)); % left peak marker position
    plot(X(pos_L(1:NtopL)),dy,'k^','markerfacecolor',[1 0 0]); % Markers
    [~,pos_R]=findpeaks(-Y,'MINPEAKHEIGHT',min_R,'MINPEAKDISTANCE',splitt);
    NtopR=max(size(pos_R)); % right peak marker position
    plot(X(pos_R(1:NtopR)),dy,'kv','markerfacecolor',[0 1 0]); % Markers
    fprintf('Current upper/lower tresholds = %3.2f %3.2f:\n',vakt)
    vres(1)=input('Reset upper treshold,(0 if OK):');
    vres(2)=input('Reset lower treshold,(0 if OK):');
    if max(vres)==0
        vakt(1:2)=0;
        hold off
    else
        vakt=vres;
    end
    close (3);
end
pos_L=sort(pos_L);pos_R=sort(pos_R); % markers in ascending order
width=X(pos_R)-X(pos_L);             % width between markers
centre=(x(pos_R)+x(pos_L))/2;        % centre between markers
Linjer=min(max(NtopR),max(NtopL));
wT(1:Linjer)=0;
for L=1:Linjer                        % widths by parabolic fit
    wL=parabel(X,Y,pos_L(L));  wR=parabel(X,Y,pos_R(L));  wT(L)=wR-wL;
end
for L=1:Linjer                         % O2 pressures
    O2=(tryck(width(L))+tryck(wT(L)))/2;
    dO2=abs(tryck(width(L))-tryck(wT(L)))/2;
    fprintf('[pO2] = %5.2f +- %5.2f mm Hg, ', O2,dO2);
    fprintf('Width(exp.)  = %6.3f G, ', width(L));   % exp width
    fprintf('Width(fit)   = %6.3f G \n', wT(L));     % simulated width
end
ymod(1:Linjer,1:nx)=0;
for L=1:Linjer
    X=x-centre(L);
    [ymod(L,:),~,~] = Simuline(X,width(L),L_G);      % simulated line
end
a=ymod'\Y;
T=ymod'*a;                            % Composed spectrum
figure;
plot(x,T,'r',x,Y,'LineWidth',1);      % Exp. & calc. spectra
xlabel('Field (G)'); ylabel('Intensity');
title('Experimental (blue) and fitted (red) lines')
hold on
plot(x(pos_L),0,'k^','markerfacecolor',[1 0 0]);  % Left  markers
plot(x(pos_R),0,'kv','markerfacecolor',[0 1 0]);  % Right markers
end
```

```matlab
function [z,brus,val] = Y_filt(X,Y,Tc,m) % Filter selection
% IN
% X: field, Y: amplitude
% Tc: Time constant (ms) for RC-filter
% m: Averaging over 2m+1 points with moving average filter
% OUT
% z: filtered amplitude
% brus: noise level
% val: flat/1; binomial/2; Savitzky-Golay/3; RC/4; none/5 filter selection
[bas,~] = baseline(X,Y);                 % baseline
nX=max(size(X));                         % number of field points
Xtext=X(1)-(X(nX)-X(1))*.1;              % text position
y_B=Y-bas;
y(1,:) = datasmooth(y_B,m,'flat');       % flat moving average
y(2,:) = datasmooth(y_B,m,'binom');      % binomial moving average
y(3,:) = datasmooth(y_B,m,'savgol',2);   % Savitzky-Golay moving average
y_rc = rcfilt(y_B,1,Tc,'up');            % RC filter
y(4,:) = rcfilt(y_rc,1,Tc,'down');
y(5,:) = y_B-mean(y_B);                  % original signal
d=(max(y(5,:))-min(y(5,:)))/4;
figure(1)
plot(X,y(1,:)+d,X,y(2,:)+2*d,X,y(3,:)+3*d,X,y(4,:)+4*d,X,y(5,:)+5*d)
xlabel('Field (G)');ylabel('Intensity');
title('Filters: flat/1; binomial/2; Savitzky-Golay/3; RC/4; none/5');
text(Xtext,d,'flat/1');text(Xtext,2*d,'binom/2');
text(Xtext,3*d,'savgol/3');text(Xtext,4*d,'RC/4');text(Xtext,5*d,'raw/5');
val=input('Filters: flat/1; binomial/2; Savitzky-Golay/3; RC/4; none/5:');
if 6<=val || val<=0
    val=4; %RC filter by default
    fprintf('Wrong filter # was entered, RC filter will be used\n');
end
nx=max(size(X));
z(1:nx)=0;
z(:)=y(val,:)/(max(y(val,:))-min(y(val,:)));% normalized signal
[bas,brus] = baseline(X,z);                    % baseline & noise
z=z'-bas;
end

function [bas,brus] = baseline(X,Y)           % bas= baseline  of spectrum
% IN
% X: field, Y: amplitude
% OUT
% bas: baseline
% brus: noise level
nx=max(size(Y));nb=fix(nx/30);                % nb= # of data for base-line
y(1:nb)=Y(1:nb); y(nb+1:2*nb)=Y(nx-nb+1:nx); % y-values used for linefit
x(1:nb)=X(1:nb); x(nb+1:2*nb)=X(nx-nb+1:nx); % x-range used for linefit
brus=max(y)-min(y);                           % noise
Bf=[ones(2*nb,1) x'];                         % Basis
c=Bf\y';
bas=c(1)+c(2)*X;                              % base-line
end

function [U,wT,Sh] = Simuline(X,wX,a) % U=simulated line, wT=width, Sh=shape
% IN
% X: field values
% wX: exp. linewidth
% a: Lorentz/Gauss linewidth ratio
% OUT
% U: calculated amplitude
```

```
% wT: calculated width
% Sh: a=0/Gauss a=inf/Lorentz/else Voigt shape
nx=size(X,1); u(1:nx)=0;U(1:nx)=0;  % nx=number of field points
np=5;                                % # of data to obtain peak-peak width
if a==0
    Sh='(Gauss)'; f=1.4142135626/wX;           % Gauss-shape
    u=exp(-(f*X).^2);                          % absorption
    U=-f*X'.*u';                               % derivative
elseif a==inf
    Sh='(Lorentz)'; f=1.154700538/wX;          % Lorentz-shape
    u=ones(nx,1)./(1+(f*X).^2);                % absorption
    U=-f*X'.*u'.^2;                            % derivative
else
    Sh='(Voigt)';                              % Voigt-shape
    f=2*(0.5436*a+sqrt(0.2166*a^2+1));         % approximate linewidth
    for n=1:nx;
        u(n)=Voigt7_G(f*X(n),a);               % absorption
    end
    for n=2:nx-1; U(n)=u(n+1)-u(n-1); end;     % derivative
    U(1)=U(2); U(nx)=U(nx-1);
    wL = topp(X,U,np); wR = topp(X,-U,np); wT=wR-wL;
    for n=1:nx; u(n)=Voigt7_G(f*wT*X(n)/wX,a); end
    for n=2:nx-1; U(n)=u(n+1)-u(n-1); end;     % corrected derivative
    U(1)=U(2); U(nx)=U(nx-1);
end
wL = topp(X,U,np);wR = topp(X,-U,np);          % positive & negative peaks
wT=wR-wL;                                      % Simulated peak-peak width
end

function re=Voigt7_G(z,y) % Gautschi's algorithm for error function
% IN
% z=field, y=Lorentz/Gauss line width ratio
% y=0: Gauss line, y=inf: Lorentz line, 0<y<inf: Voigt line
% OUT
% re: Voigt function = real part of error function
% References:
% W. Gautschi, SIAM J. Numer:Anal.,7,187 (1970)
% Algol: W. Gautschi, Comm. ACM.,12(11) (1969), 635
% Fortran: J. Maruani (unpublished)
x=abs(z);
if (y<4.29 && x<5.33)
    s=(1-y/4.29)*sqrt(1-x^2/28.4089); h=1.6*s;
    h2=2*h; capn=fix(6+23*s); nu=fix(9+21*s);
else
    h=0; capn=0; nu=8;
end
if h>0
    lambda=h2^capn;
end
r1=0;r2=0;s1=0;s2=0;
n=nu;
while n>=0
    t1=y+h+(n+1)*r1; t2=x-(n+1)*r2; c=.5/(t1^2+t2^2); r1=c*t1; r2=c*t2;
    if h>0 && n<=capn
        t1=lambda+s1; s1=r1*t1-r2*s2; s2=r2*t1+r1*s2; lambda=lambda/h2;
    end
    n=n-1;
end
if h==0||lambda==0
    re=1.12837916709551*r1;
else
```

```
    re=1.12837916709551*s1;
end
end

function [x0,y0] = parabel(x,y,ix) % parabolic fit of line-peak
% IN
%(x,y)=spectrum, nx= point # near line-peak
% OUT
% x0,y0: peak position
ip=11; % # of points in fit
i2=round((ip+1)/2);
if ix>=i2
    X(1:ip)=x(ix-i2+1:ix+i2-1);Y(1:ip)=y(ix-i2+1:ix+i2-1);
    Bf=[ones(ip,1) X' X'.^2];          % Basis functions
    c=Bf\Y';
    x0=-c(2)/(2*c(3));                 % x0 at dy/dx=0
    y0=c(1)+c(2)*x0 +c(3)*x0.^2;
else
    fprintf('Analysis stopped because ix = %i in *parabel*\n',ix)
end
end

function [w] = topp(x,U,np) %parabolic fit to obtain x=w at dU/dx = 0
% IN
%(x,U)=spectrum, np= number of points
% OUT
% w: peak position
% U = c(1)+c(2)*(X-X(1))+c(3)*(X-X(1))*(X-X(2))  (1)
[~,I]=max(U);ix=I(1);w=x(ix);       % approximate peak position
if ix>(np-1)/2
    Y(1:np)=U(ix-(np-1)/2:ix+(np-1)/2);X(1:np)=x(ix-(np-1)/2:ix+(np-1)/2);
    A=[ones(np,1) (X'-X(1)) (X'-X(1)).*(X'-X(2))]; %Basis functions in (1)
    c=A\Y';
    w=(X(1)+X(2))/2-c(2)/(2*c(3)); % refined peak position  at dU/dX =0
else
    disp(' Refined peak position not found in*topp*');
end
end

function [O2_exp] = tryck(exp_width)  % [pO2] from measured LiPc EPR width
%IN
% exp_width: linewidth (G)
% Out
% O2_exp: O2 pressure (mm Hg)
O2_cal=[0 5 21]*7.60;                 % O2 pressure
w_cal=[0.03 0.24 0.86] ;              % LiPc peak-peak linewidths
nx=size(O2_cal,2);
Bf=[ones(nx,1) w_cal' w_cal'.^2];     % Basis functions
c=Bf\O2_cal';
O2_exp=c(1)+c(2)*exp_width' +c(3)*exp_width'.^2;
End
```

## 10. Summary

Software has been developed to obtain the oxygen concentration, ($pO_2$) by EPR measurements *in vivo*. The experimental EPR spectra were digitally filtered to improve the SNR prior to line-width measurement of the used LiPc probes. Tools available in Matlab and Easyspin [12] were employed to automatically compute the widths, from which the oxygen concentration could be calculated.

**References**


1. Dasu A, Toma-Dasu I (2008) Vascular oxygen content and the tissue oxygenation - a theoretical analysis. Medical Physics 35:539-545
2. Eriksen JG, Horsman MR, (2006) Tumour hypoxia - A characteristic feature with a complex molecular background. Radiother. Oncol. 81:119-121.
3. Wijffels KI, Kaanders JH, Rijken PF, Bussink J, van den Hoogen FJ, et al. (2000) Vascular architecture and hypoxic profiles in human head and neck squamous cell carcinomas. Br. J. Cancer 83:674–683.
4. Taylor NJ, Baddeley H, Goodchild KA, Powell MEB, Thoumine M, Culver LA, et al. (2001) BOLD MRI of human tumor oxygenation during carbogen breathing. J. Magn. Reson. Imaging 14:156–63.
5. Liu KJ, Gast P, Moussavi M, Norby SW, Vahidi N, et al. (1993) Lithium phthalocyanine: A probe for electron paramagnetic resonance oximetry in viable biological systems. PNAS 90:5438-5442
6. Swartz HM, Williams BB, Zaki BI, Hartford AC, Jarvis LA, et al. (2014) Clinical EPR: Unique opportunities and some challenges. Acad. Radiol. 21:197–206
7. Epel B, Redler G, Halpern HJ (2014) How in vivo EPR measures and images oxygen. Advances in experimental medicine and biology 812:113-119.
8. Franzén S, Pihl L, Khan N, Palm F, Gustafsson H (2014) Repetitive measurements of intrarenal oxygenation in vivo using L-band Electron Paramagnetic Resonance. Advances in Experimental Medicine and Biology 812:135-141.
9. Weil JA, Bolton JR (2007) Electron paramagnetic resonance: Elementary theory and practical applications. Wiley, Hoboken NJ.
10. Robinson BH, Mailer C, Reese AW (1998) Linewidth Analysis of Spin Labels in Liquids: I. Theory and Data Analysis. J. Magn. Reson. 138:199–209
11. Cochrane CJ, Lenahan PM (2008) Real time exponentially weighted recursive least squares adaptive signal averaging for enhancing the sensitivity of continuous wave magnetic resonance, J. Magn. Reson. 195:17-22
12. (a) Stoll S, Schweiger A (2006) EasySpin, a comprehensive software package for spectral simulation and analysis in EPR. J Magn Reson. 178:42–55; (b) http://www.easyspin.org/
13. Gustafsson H, Kale A, Dasu A, Lund A, Edqvist P-H, Roberg K, EPR Oximetry of Cetuximab-Treated Head-and-Neck Tumours in a Mouse Model. Cell Biochem Biophys, in press, DOI 10.1007/s12013-017-0814-5
14. Gustafsson H, Lund A, EPR_width_RC.m: Software for in vivo oximetry using LiPc EPR Probes In manuscript.
15. Khan N, Mupparaju S, Hou H, Williams BB, Swartz H (2012) Repeated assessment of orthotopic glioma pO(2) by multi-site EPR oximetry: a technique with the potential to guide therapeutic optimization by repeated measurements of oxygen. J Neurosci Methods 204:111–117
16. Gautschi W, (a) (1969) Algorithm 363. Complex error function, Comm. ACM 12:635; (b) (1970) Efficient computation of the complex error function, SIAM Journal on Numerical Analysis 7:187-198
